# Cerebral Microbleed Burden in Ischemic Stroke Patients on Aspirin: Prospective Cohort of Intracranial Hemorrhage

**DOI:** 10.3389/fneur.2021.742899

**Published:** 2021-10-28

**Authors:** Chong-xi Xu, Hui Xu, Tong Yi, Xing-yang Yi, Jun-peng Ma

**Affiliations:** ^1^Department of Neurosurgery, West China Hospital of Sichuan University, Chengdu, China; ^2^Department of Neurosurgery, The Second People's Hospital of Liangshan Yi Autonomous Prefecture, Liangshan, China; ^3^Department of Neurology, The Second People's Hospital of Deyang City, Deyang, China; ^4^Department of Neurology, People's Hospital of Deyang City, Deyang, China

**Keywords:** cerebral microbleeds, ischemic stroke, intracranial hemorrhage, susceptibility-weighted imaging, aspirin

## Abstract

**Objective:** This investigation aimed at studying the prevalence of cerebral microbleeds (CMBs), including risk factors and the correlation of CMBs to ischemic stroke (IS) patient end results.

**Methods:** Four hundred and fifty-nine acute IS cases were recruited between April 2014 and December 2016. Cerebral microbleeds were analyzed using susceptibility-weighted imaging (SWI) brain MRI scan. The enrolled patients with acute IS were followed up for 12–24 months, with a median follow-up time of 19 months. The follow-up endpoint events including recurrent ischemic stroke (RIS), intracranial hemorrhage (ICH), transient ischemic attack (TIA), mortality, and cardiovascular events. The associations between vascular risk factors and CMBs in IS patients were analyzed using univariate and multivariate logistic regression analysis. Cox regression model was employed for evaluating CMB impact on clinical outcome.

**Results:** Among 459 enrolled patients, 187 (40.7%) had CMBs and 272 (59.2%) had no CMB. In comparison with patients with no CMBs, age was higher and hypertension was more frequent in patients with CMBs. Multivariate logistic regression analyses revealed age and hypertension were independently associated with the presence of CMBs. Among the patient cohort, 450 cases completed the follow-up. During the follow-up period, 22 (4.9%) of patients developed ICH, 12 (2.7%) developed TIA, 68 (15.1%) developed RIS, cardiovascular events occurred in 20 (4.44%), and 13 (2.89%) cases were mortalities. Compared with patients without CMBs, IS patients with CMBs have an increased prevalence of ICH (*p* < 0.05). However, no statistically valid variations regarding other outcome incidences between both groups was identified (*p* > 0.05). The incidence of ICH was elevated in tandem with elevations in number of CMBs. Following adjusting for age, multivariate Cox proportional-hazards regression analysis revealed that CMBs ≥10 were independent predictors of ICH in acute IS patients.

**Conclusion:** Age and hypertension are independently associated with the presence of CMBs. Intracranial hemorrhage incidence rate was increased with the number of CMBs, and the number of CMBs ≥10 were independent predictors of ICH in acute stroke patients.

## Introduction

The concept of cerebral microbleeds (CMBs) was first proposed in the mid-1990s, and it is caused by the deposition of hemosiderin in the brain caused by the leakage of red blood cells from small blood vessels (1). It is one of the typical imaging manifestations of cerebral small vessel diseases (2). Risk factors for the presence of CMBs remain unclear. Investigations demonstrated that CMBs, caused by varying mechanisms, can occur in any location of the brain (3). Recent systematic reviews demonstrated that increased number of CMBs are linked to exacerbated risks for intracranial hemorrhage/ischemic stroke (ICH/IS), and the presence of CMBs may play a key role in vascular cognitive impairment (4, 5).

Magnetic resonance gradient echo T2-weighted imaging (T2^*^ WI) (GE-T2^*^ WI) is a highly sensitive technique for detecting CMBs, which can detect hemosiderin deposition (of several millimeters in size) within the brain parenchyma. Recently, studies have shown that three-dimensional fast field echo susceptibility-weighted imaging (3D-FFESWI), susceptibility-weighted imaging (SWI) demonstrates superior sensitivity and specificity in detecting CMB regions, in comparison with GE-T2^*^ WI (5, 6). Cerebral microbleed anatomical scoring scale (7) and CMB observation scale (8) are often used to evaluate CMB distribution and size. According to the number of detected CMBs, they are divided into grade 0 (none), grade 1 (CMBs: 1–4), grade 2 (CMBs: 5–9), and grade 3 (CMs ≥10) (9).

It is established that CMBs are widespread in the elderly population (10), being linked to age, hypertension, stroke history, fibrinogen, and blood pressure (11). The incidence of presence of CMBs is increasing with age, and it is obviously correlated with IS (11, 12). There are also reports that CMBs occur in coagulopathy and anticoagulation therapy (13, 14).

Several investigations highlighted that CMBs are linked to exacerbated risks for imminent ICH, particularly patients treated with aspirin (15). On the other hand, CMBs could additionally be highly correlated to recurrence of IS and poor functional prognosis post-acute IS (4). However, there are some inconsistent results. Fiehler et al. (16) showed that the presence of CMBs increased the incidence of post-IS ICH, although ICH was a low-probability event. Another study revealed that the risk of ICH did not increase within aspirin-treated patients, in comparison with IS/CMB positive patients with no aspirin treatments (17). However, Soo et al. (18) reported that IS/CMB positive patients do incur additional risks in developing sICH following antiplatelet aggregation drugs. So whether ischemic stroke (IS) patients with high burden of CMBs receiving aspirin treatment lead to poor outcomes remains controversial.

So in general, within IS patients, high burden of CMBs (CMB number ≥5) tend to predict ICH (9) and IS (19), although the risk/benefit ratio of antiplatelet treatments within IS cases bearing CMBs remains unclear.

Consequently, this study focuses on those IS patients receiving antiplatelet therapy and this investigation consisted of two phases. Initial focus was set on the prevalence and risk factors for the presence of CMBs in IS clinical cases. Second, a prospective cohort study was performed to clarify the IS recurrence rate, ICH incidence rate, together with the incidence rate for other vascular events throughout the follow-up timeframe. Evaluation of risk/benefit ratio for antiplatelet treatments in IS/CMB positive cases was also performed.

## Materials and Methods

### Investigation Patient Cohorts

Between April 2014 and December 2016, 459 patients with IS were recruited. Patients with IS were validated through brain MRI scans, followed by admittance to the People's Hospital of Deyang City and Second People's Hospital of Deyang City within 48 h following the onset of symptoms. Inclusion criteria: (1) patients with IS confirmed by symptoms and imaging findings; (2) patients who have passed the thrombolytic time window or choose to refuse thrombolytic therapy. Exclusion criteria were as follows: (1) patients contraindicated for MRI scan; (2) brain tumors, arteriovenous malformations, and ruptured hemangioma; (3) thrombolytic therapy; (4) unwillingness to participate in this study. This study was accepted by the hospitals' Ethics Committee. Informed consent was obtained for all patient cases.

### Brain MRI and Defining Criteria for CMB Events

Patients with IS were scanned using a 3-T MRI scanner. Cerebral microbleeds were detected using SWI (20). Cerebral microbleeds were classified as aged, silent foci of signal absence/hypo-intensity on SWI, measuring approximately 2–10 mm diameter. According to previous studies, the location of CMB events was scored through employment of the microbleed Anatomical Rating Scale (1, 7), with burden (numbers) stratified as grade 0 (none), grade 1 (1–4), grade 2 (5–9), and grade 3 (≥10).

### Clinical Variables

Demographics (age, sex) and relevant vascular-based risk parameter data were collected, including atrial fibrillation, coronary heart conditions, history of stroke hypertension, smoking, and diabetes mellitus. Fasting blood samples were measured for glucose, total plasma cholesterol (TC), triglycerides (TG), low-density lipoprotein cholesterol, high-density lipoprotein cholesterol, and homocysteine blood levels. Hyperlipidemia was deemed to be TC >200 mg/dl, TG >180 mg/dl, or use of lipid-lowering drugs. Stroke severity evaluation was performed by a certified member of the stroke team, using the National Institutes of Health Stroke Scale (NIHSS) once the individual patient was admitted.

### Prospective Cohort Study

Following hospital discharge, all patients were followed up by a research nurse or physician on a quarterly basis, for 12–24 months (median = 19 months). Clinical cases within this study all received standardized medical care according to guidelines for the prevention of stroke in stroke/transient ischemic attack (TIA) patients (21). During the follow-up period, the entire cohort was assessed for multiple clinical outcomes: (1) recurring stroke (ischemic, hemorrhagic, transient TIA); (2) mortality (vascular and nonvascular); (3) cardiovascular events (myocardial infarction, angina). The definition of recurrent stroke required a sudden new neurological deficit fitting the definition of IS or ICH by the physical and imaging examinations.

### Statistical Analysis

SPSS V.21.0 software was employed, with continuous variables expressed as mean ± SD. Normality status of numerical variables was assessed through the Kolmogorov–Smirnov test. Normal-distribution variables were compared through single-pair two-tailed independent group *t*-test. Non-normal-distribution variables were comparatively analyzed through a Mann–Whitney test. Categorical variables were expressed as percentage rates and compared by χ^2^-test, or (if predicted variable frequencies were minimal) Fisher's exact tests were performed. The clinical data and baseline characteristics for patients with/without CMBs were investigated through univariate analysis methodologies. Multivariate logistic stepwise regression analysis was employed for exploring risk factors of CMBs, and multivariate Cox regression model was employed for evaluating the impact of CMBs on clinical outcome. The variables inputted for multivariate logistic stepwise regression and Cox regression model were variables with a *p*-value <0.1 (by univariate analysis). Data outcomes of the Cox model were listed as the hazard ratio (HR), with a 95% CI. Survival function estimates for ICH, according to the quantities of CMB (no CMBs, 1–4 CMBs, 5–9 CMBs, ≥10 CMBs), were analyzed through Kaplan–Meier curve/log-rank test analyses. Survival curves were truncated at 24 months. Datasets with a *p*-value of <0.05 were deemed to be statistically significant.

## Results

### Incidence Rate and Risk Factors for CMBs Within Acute IS Cases

Of the 459 patients with acute IS, 187 (40.7%) had CMBs and 272 (59.2%) had no CMBs (Table 1). The age was older and hypertension was more frequent in IS patients with CMBs than those without CMBs (Table 1). Multivariate logistic stepwise regression analyses revealed age and hypertension to be independently associated with the presence of CMBs in IS patients.

**Table 1:** Patient demographics and medical data.

**Variable**	**CMBS (*n* = 187)**	**No CMBS (*n* = 272)**	***p*-Value**
Age, years (means ± SD)	69.02 ± 11.338	66.13 ± 12.022	0.010
Male, *n* (%)	133 (71.9)	181 (66.5)	0.226
History of IS (%)	28 (15.1)	39 (14.3)	0.813
History of ICH (%)	8 (4.3)	6 (2.2)	0.197
Smoking (%)	99 (53.5)	125 (46)	0.113
Hypertension (%)	120 (64.8)	147 (54)	0.021
Diabetes mellitus (%)	44 (23.8)	69 (25.4)	0.700
Dyslipidemia (%)	4 (2.2)	9 (3.3)	0.662
Atrial fibrillation (%)	10 (5.4)	13 (4.8)	0.764
Valvular heart disease (%)	5 (2.7)	10 (3.6)	0.566
NIHSS	5.36 ± 5.268	4.84 ± 5.090	0.151
Triglycerides (mmol/L)	1.451 ± 0.984	1.428 ± 1.101	0.796
Total plasma cholesterol (mmol/L)	4.293 ± 0.894	4.264 ± 1.024	0.050
Low-density lipoprotein cholesterol (mmol/L) (means ± SD)	2.422 ± 0.758	2.427 ± 1.099	0.193
High-density lipoprotein cholesterol (mmol/L) (means ± SD)	1.1311 ± 0.352	2.046 ± 10.606	0.184

*NIHSS, National Institute of Health Stroke Scale; IS, ischemic stroke; ICH, intracranial hemorrhage*.

### Antiplatelet Therapy

Stemming from 459 patients, 173 cases were managed using aspirin alone, 3 were managed using clopidogrel therapy alone, and 283 cases were treated with aspirin and clopidogrel within 21 days from onset of symptoms, followed by aspirin thereafter. Almost all patients were treated with aspirin (456 cases−99%).

### Outcomes

Stemming from the 459-patient cohort, 9 cases were lost from follow-up due to incorrect patient contact details although 450 cases completed the follow-up. The rate of loss to follow-up was 2.0%.

**Figure 1:**
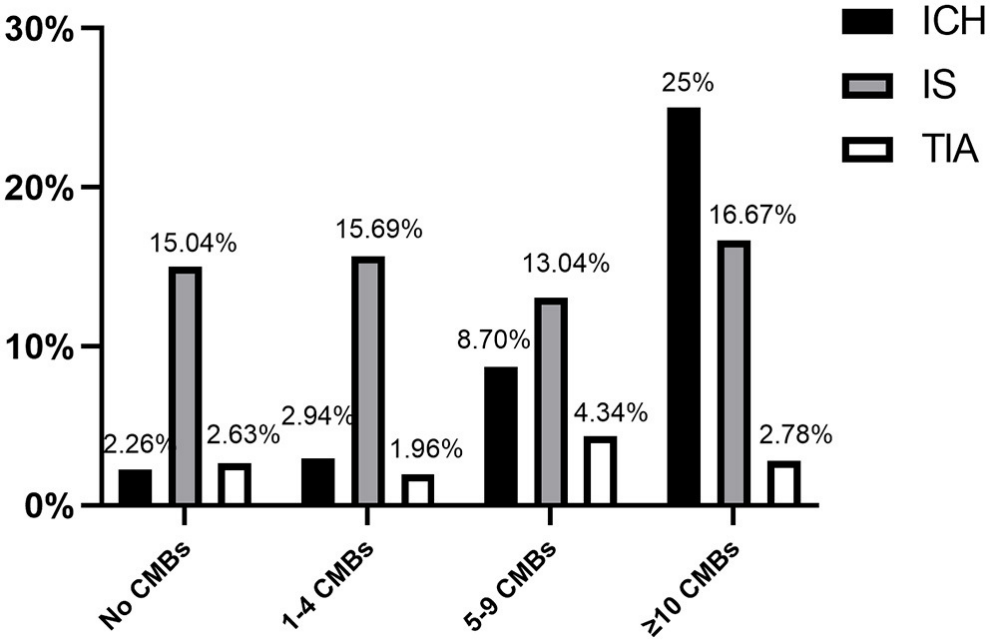
Main study endpoints, separated based on number of CMB events (ICH, intracerebral hemorrhage; IS, ischemic stroke recurrence; TIA, transient ischemic attack).

During the follow-up period, 22 (4.9%) patients developed ICH, 12 (2.7%) patients developed TIA, 68 (15.1%) patients developed recurrent ischemic stroke (RIS), cardiovascular events occurred in 20 (4.44%), and 13 (2.89%) cases led to a mortality due to cardiovascular or cerebrovascular event. Outcomes in patients with/without CMBs are displayed in Table 2. The incidence of ICH was higher in patients with CMBs than those without CMBs [8.70% (16/184) vs. 2.26% (6/266), *p* = 0.003] (Table 2; Figure 1). Notwithstanding, there were no significant differences in incidence of RIS, cardiovascular events, TIA, and mortality between patients with and without CMBs (Table 2; Figure 1).

**Table 2:** Outcomes in patients with and without CMB events.

**Outcomes**	**Patients with CMBs**	**Patients without CMBs**
ICH	8.70% (16/184)	2.26% (6/266)
IS	15.22% (28/184)	15.04% (40/266)
TIA	2.72% (5/184)	2.63% (7/266)
Mortality	2.72% (5/184)	3.01% (8/266)

*CMB, cerebral microbleed; ICH, intracranial hemorrhage; IS, ischemic stroke; TIA, transient ischemic attack*.

Outcomes such as ICH, IS, and TIA were stratified according to the numbers of CMBs (i.e., 1–4 CMBs, 5–9 CMBs, and ≥10 CMBs) and patients with no CMBs (Figure 1). Intracranial hemorrhage events had a directly proportional correlation with frequency of the number of CMBs: 2.9% in patients with 1–4 CMBs, 8.7% with 5–9 CMBs, and 25% with ≥10 CMBs. However, the incidence of RIS and TIA did not increase with the number of CMBs (Figure 1).

**Figure 2:**
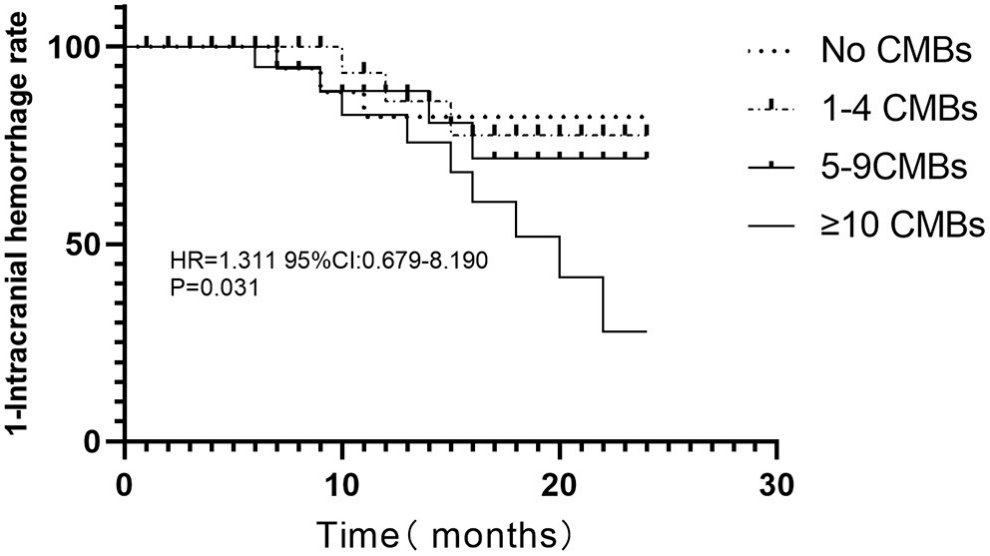
Kaplan–Meier curve for consequent intracerebral bleeding (log-rank test).

Univariate Cox proportional-hazards regression showed that age, CMB occurrence, and the CMBs ≥10 were significantly associated with ICH (*p* < 0.05, Table 3). According to previous research (18) and our study results, age deemed to be linked with CMBs and additional risk for ICH. Multivariate Cox proportional-hazards regression analysis revealed that CMB events ≥10 were independent predictors of ICH post-acute IS, after adjusting for age (Table 3). The Kaplan–Meier curve revealed intimate correlation between the number of CMBs and ICH development risks (Figure 2).

**Table 3:** Univariate Cox regression analyses for consequent ICH and multivariate Cox regression analyses for ≥10 CMB events in predicting subsequent ICH (adjusted by age).

**Variable**	**Univariate analysis**				**Multivariate analysis**			
	***p*-Value**	**HR**	**95% CI**		***p*-Value**	**HR**	**95% CI**	
			**Lower**	**Upper**			**Lower**	**Upper**
Presence of CMBs	0.007	17.00	2.193	131.785				
1–4 CMBs	0.164	0.025	0.00	4.490				
5–9 CMBs	0.729	1.314	0.280	6.160				
≥10 CMBs	<0.001	26.311	5.679	121.90	0.031	1.311	0.679	8.190
Male	0.062	0.336	0.106	1.058				
Age	0.025	0.962	0.930	0.995	0.041	2.72	1.013	3.210
History of coronary heart disease	0.478	0.046	0.000	230.637				
Dyslipidemia	0.563	21.247	0.001	670986				
History of ischemic stroke	0.991	1.007	0.296	3.421				
History of cerebral hemorrhage	0.884	1.161	0.155	8.727				
Atrial fibrillation	0.812	0.783	0.105	5.864				
Valvular heart disease	0.764	1.360	0.182	10.148				
Diabetes mellitus	0.506	0.691	0.232	2.055				
Hypertension	0.301	0.620	0.122	3.472				

*HR, hazard ratio*.

Univariate Cox proportional-hazards regression revealed that age, smoking, and hypertension were significantly associated with RIS (Table 4). Multiple Cox regression analysis showed that age, smoking, and hypertension were also independent predictors for RIS. However, CMBs were not significantly linked to RIS (Table 4).

**Table 4:** Univariate Cox regression analyses and multivariate Cox regression analyses for recurrence of ischemic stroke.

**Variable**	**Univariate analysis**				**Multivariate analysis**			
	***p*-value**	**HR**	**95% CI**		***p*-value**	**HR**	**95% CI**	
			**Lower**	**Upper**			**Lower**	**Upper**
Presence of CMBs	0.341	5.120	3.113	80.785				
Male	0.821	6.231	2.323	11.038				
Age	0.011	2.332	1.021	3.112	0.031	1.082	0.321	6.332
Smoking	0.041	0.321	0.012	2.321	0.021	0.672	0.021	3.991
Hypertension	0.018	0.620	0.122	3.472	0.019	2.113	1.001	4.902
History of coronary heart disease	0.812	2.122	0.000	122.23				
Dyslipidemia	0.912	18.053	1.223	40.003				
History of ischemic stroke	0.061	2.312	0.123	4.221				
History of cerebral hemorrhage	0.663	2.112	1.003	7.883				
Atrial fibrillation	0.678	2.337	0.112	4.862				
Valvular heart disease	0.882	1.221	1.001	6.992				
Diabetes mellitus	0.081	0.882	0.001	1.223				

*HR, hazard ratio*.

## Discussion

The pathogenesis of occurrence of CMBs is not yet fully understood. Blood–brain barrier damage, endothelial cell dysfunction, neovascularization, apolipoprotein E (apoE) ε4 genotype, and inflammation are intimately correlated to CMB etiology (22, 23). The incidence, location, and number of CMBs are affected by many parameters, including hypertension, diabetes, hypercholesterolemia, smoking, and obesity (11). Such risk factors are highly related to micro-circulatory vascular disease, consequently exacerbating CMB occurrence risks. Presently, research on CMBs are mainly based on the links between cerebral microcirculatory vascular disease and stroke, particularly regarding IS. Poels et al. (12) demonstrated CMB incidence in acute IS patients aged >80 years was as high as 35.7%, which was significantly higher than within patients aged ≤ 80 years. These results were consistent with our current results.

Presently, the prevention and treatment of CMBs is still under exploration. The occurrence of CMBs is closely related to the risk of cardiovascular and cerebrovascular diseases. Therefore, this is crucial to the prevention and treatment of occurrence of CMBs. This investigation revealed that age was higher within IS patients with CMBs. Hypertension was also more prevalent within the CMB positive group. Multivariable logistic regression analysis results demonstrated the occurrence of CMBs to be intimately correlated to age/hypertension. Therefore, for acute IS patients with increased age or hypertension, imaging examinations should be performed in a timely manner for awareness of CMB occurrence.

Lee et al. (9) speculate that the presence of CMBs is highly related to ICH. Also, the presence of CMBs exacerbates ICH incidence within IS patients undergoing thrombolytic therapy (24). However, whether IS patients with high burden of CMBs (CMBs ≥10) receiving aspirin treatment lead to poor outcomes remains controversial. It is known that oral antiplatelet drugs in IS cases could effectively lower the possibility for IS recurrence. However, selected studies have shown that antiplatelet drugs (aspirin alone or the combination of two antiplatelet drugs) increases the incidence of CMBs (25). A systematic review and meta-analysis showed that risk of ICH was higher in IS patients with CMBs who are receiving antiplatelet therapy compared with the patients without CMBs, and the number of CMBs was significantly associated with subsequent ICH after the use of antiplatelet agents, especially when CMBs >5 (26).

In comparison with other antiplatelet drugs such as clopidogrel, cilostazol, and ticlopidine, aspirin has the highest correlation with antiplatelet-associated hemorrhages (27). Also, research showed that there is no difference between the use of aspirin alone or the combination of two antiplatelet drugs for increasing the incidence of CMB-related ICH (28). Regarding IS patients with CMBs, the current guidelines do not provide opinions on the safety of antiplatelet therapy. Lau et al. (15) suggested that antiplatelet drugs cannot be stopped within 1 year of onset for these patients. However, they also pointed out that the risk of ICH may outweigh any benefit thereafter for IS patients with CMBs ≥5. Therefore, whether post-acute phase gradual discontinuation of antiplatelet drugs can bring benefits or otherwise still requires further research.

As shown in this study, in the case of almost all patients (99%) receiving aspirin treatment, 68 (15.11%) patients developed RIS and 22 patients developed subsequent ICH (4.89%). The risk of RIS is higher than the incidence of subsequent ICH during the follow-up period. There was no significant difference in incidence of RIS between patients with and without CMBs. Smoking, hypertension, and age were independent predictors for RIS. CMBs ≥10 were independent predictors of subsequent ICH after adjusting for age. Our current results were consistent with other previous studies, which is that CMBs is a relevant and accurate predictor of hemorrhagic transformations in acute IS (15, 18, 29). Our study also showed that although IS patients with high burden of CMBs have a higher probability of ICH, the recurrence rate of IS is still much higher than the probability of cerebral hemorrhage in all patients regardless of whether they have CMBs, and this result is also consistent with previous studies (30). It is indicating that antiplatelet treatment is still necessary for these patients. However, the ICH occurrence risks were highly exacerbated for IS patients bearing CMBs ≥10, which means that the ICH occurrence risks overwhelmed benefits for these patients treated with antiplatelet drugs. However, further well-designed studies are warranted to replicate this finding.

This study does have a set of limitations. First, this investigation did not include patients on thrombolytic therapy, and we did not know the occurrence of ICH post-thrombolytic therapy in patients with CMBs. According to Fiehler's research (16), IS cases having CMBs tend not to raise the possibility of ICH occurrence post-thrombolysis. However, the study by Tsivgoulis indicated that acute IS patients with CMBs have an increased danger of incurring sICH post-intravenous thrombolysis, in comparison with CMB-free cases, which means that CMBs are being intimately correlated to the possibility of incurring post-thrombolytic ICH. Moreover, they also indicated that high CMB burden (CMB number ≥10) may be included in individual risk stratification scores predicting sICH risk following IVT for AIS (24). So the rapid progression of CMBs post-thrombolysis can be one of the causes of ICH. Second, due to condition requirements, most of our patients were treated with aspirin, and there was no comparison of the prognosis for various antiplatelet drugs. Third, due to the limited sample size and two-center design of this study, our findings must be confirmed with larger, multi-center studies.

## Conclusion

Age and hypertension are independently associated with CMBs in IS patients. Intracranial hemorrhage prevalence was exacerbated within patients with the number of CMBs, while CMBs ≥10 were independent predictors of ICH on follow-up assessments.

## Data Availability Statement

The original contributions presented in the study are included in the article/supplementary material, further inquiries can be directed to the corresponding author/s.

## Ethics Statement

The studies involving human participants were reviewed and approved by the Subject of Deyang Science and Technology Bureau, China. The patients/participants provided their written informed consent to participate in this study.

## Author Contributions

C-xX and HX contributed to the drafting. J-pM, TY, and X-yY contributed to the concept and revision of the article. All authors have read and approved the final version to be published.

## Funding

This study was supported by the Subject of Deyang Science and Technology Bureau, China (grant no. 2016SE030).

## Conflict of Interest

The authors declare that the research was conducted in the absence of any commercial or financial relationships that could be construed as a potential conflict of interest.

## Publisher's Note

All claims expressed in this article are solely those of the authors and do not necessarily represent those of their affiliated organizations, or those of the publisher, the editors and the reviewers. Any product that may be evaluated in this article, or claim that may be made by its manufacturer, is not guaranteed or endorsed by the publisher.

## Abbreviations

CMB: cerebral microbleed
IS: ischemic stroke
SWI: susceptibility-weighted imaging
RIS: recurrent ischemic stroke
ICH: intracranial hemorrhage
TIA: transient ischemic attack
GE-T2^*^ WI: gradient echo T2-weighted imaging
sICH: symptomatic cerebral hemorrhage
NIHSS: National Institutes of Health Stroke Scale
TG: triglycerides
TC: total plasma cholesterol.
